# Low Testosterone and Sperm Quality Alterations: A Prospective Study of Sperm DNA Fragmentation and Chromatin Condensation in Infertile Men

**DOI:** 10.3390/biology15030287

**Published:** 2026-02-06

**Authors:** Asmaa Serbouti, Kenza Berrada, Samy Housbane, Noureddine Louanjli, Rachid Aboutaieb

**Affiliations:** 1 Laboratory of Sexual and Reproductive Health, Faculty of Medicine and Pharmacy, Hassan II University, Casablanca 20250, Morocco; kenzaberrada2306@gmail.com (K.B.); aboutaiebrachid@gmail.com (R.A.); 2 Department of Medical Informatics, Faculty of Medicine and Pharmacy, Hassan II University Casablanca, Casablanca 20250, Morocco; s.housbane@gmail.com; 3 Labomac IVF Center and Clinical Laboratory Medicine, Casablanca 20100, Morocco; n.louanjli@gmail.com; 4 Department of Urology, CHU Ibn Rochd, Casablanca 20250, Morocco

**Keywords:** testosterone, sperm DNA fragmentation, sperm chromatin condensation, sperm parameters, infertility, spermatogenesis

## Abstract

(1) Background: Testosterone plays a key role in spermatogenesis and in maintaining semen quality and sperm DNA integrity. Consequently, reduced testosterone levels may disrupt these processes and contribute to male infertility. This study aimed to evaluate the impact of low testosterone levels on semen parameters, sperm DNA fragmentation, and chromatin condensation; (2) Methods: This was a prospective study that included 214 men aged 25–45 years undergoing infertility evaluation. Participants were classified into two groups according to serum testosterone levels: low testosterone and normal testosterone. Total testosterone was determined using electrochemiluminescence immunoassay. Semen analysis was carried out according to the WHO 2021 guidelines. The DNA fragmentation index was assessed using the TUNEL assay. The sperm decondensation index was evaluated by aniline blue staining; (3) Results: Men with low serum total testosterone levels (<2.64 ng/mL) exhibited significantly impaired semen parameters compared with those with normal testosterone levels. Serum total testosterone was positively correlated with sperm concentration (rs = 0.43, *p* < 0.001), total motility (rs = 0.20, *p* = 0.005), normal morphology (rs = 0.25, *p* < 0.001), and sperm vitality (rs = 0.173, *p* = 0.014). In contrast, testosterone levels were negatively correlated with the DNA fragmentation index (rs = −0.221, *p* = 0.0017) and the chromatin decondensation index (rs = −0.19, *p* = 0.0086). A higher proportion of pathological DFI (>15%) was observed in the low testosterone group. (4) Conclusions: These findings support the essential role of testosterone in sustaining spermatogenesis, semen quality, and sperm DNA integrity and highlight the crucial importance of testosterone assessment in the diagnosis and pathophysiological understanding of male infertility.

## Simple Summary

Male infertility represents an important public health problem worldwide. There are many factors behind male infertility, including endocrine dysfunctions. Hormones play a crucial role in the male reproductive system. In particular, testosterone, which seems to be the principal hormone implicated in testicular function, specifically regulates spermatogenesis. This study aimed to determine the relationship between testosterone and DNA fragmentation, chromatin condensation, and semen parameters. This was a prospective study that included 214 men aged 25–45 undergoing infertility evaluation. Participants were classified into two groups according to serum testosterone levels: low testosterone and normal testosterone. The results of the present study indicate that low testosterone levels are associated with increased DNA damage. Furthermore, reduced testosterone levels are linked to global alteration of the sperm quality, particularly affecting concentration, motility, morphology, and vitality of spermatozoa.

## 1. Introduction

Infertility is defined according to the World Health Organization (WHO) as the inability to achieve a clinical pregnancy after 12 months of regular, unprotected sexual intercourse [1]. It affects approximately 10–15% of couples, or about 50–80% million people worldwide [2], and the male factors are responsible for about 50% of cases [3]. Thus, it constitutes a significant public health problem [4,5]. There are many factors behind male infertility, including endocrine dysfunctions [5]. Hormones play a crucial role in the development and function of genital organs, the process of spermatogenesis, and the male reproductive system [6]. In particular, testosterone, which seems to be the principal hormone implicated in testicular function, specifically regulates spermatogenesis [7]. Testosterone is produced by Leydig cells after Luteinizing Hormone (LH) stimulation, which occurs in response to Gonadotropin-Releasing Hormone (GnRH) [6]. In men, the absence or decline of testosterone can affect spermatogenesis, leading to infertility. And low testosterone is implicated in 15% of male infertility [7,8].

In recent years, attention has shifted toward molecular markers of sperm quality, particularly sperm DNA fragmentation (SDF) [9], which is commonly assessed with the DNA fragmentation index (DFI). SDF represents single-stranded or double-stranded breaks in the genome of spermatozoa. These breaks can affect male reproductive potential. Several studies confirmed that elevated DFI has been associated with a range of negative reproductive outcomes, including reduced chances of both natural conception and success with assisted reproductive technologies, and is also associated with increased risk of recurrent pregnancy loss [9,10].

Sperm chromatin condensation (SCC) is another crucial determinant of sperm quality. And can be detected indirectly using the aniline blue staining. During spermiogenesis, the histones are replaced with protamines, and the immature chromatin conserves excess histones [11]. Protamines compact nuclear DNA into highly condensed chromatin. This compaction plays a crucial role in protecting the paternal genome during the transit of sperm through the male and female reproductive tracts, as well as during their interaction with the oocyte. Defects in chromatin condensation can lead to nuclear alterations, including SDF or DNA denaturation, which are frequently associated with male infertility [12].

Given the essential role of testosterone and the importance of genomic integrity for male fertility, evaluating the association between testosterone and sperm parameters, DNA fragmentation, and chromatin condensation may help clarify the relationships between testosterone and sperm dysfunction in infertile men.

## 2. Materials and Methods

### 2.1. Study Design and Population

This was a prospective study, conducted in the reproductive biology laboratory LABOMAC in Casablanca, Morocco. The data was collected from August 2024 to August 2025. The study population included 214 men aged 25–45 years referred to LABOMAC for infertility evaluation. All participants belonged to couples who had been trying to conceive for more than one year without success. Female partners had been clinically evaluated, and no female factor infertility was identified. The men included had no history of chronic disease (such as diabetes, hypertension, or chronic urinary tract infection), varicocele, or inflammation. And the men who had not received hormone therapy (including athletes using testosterone injections), chemo, and radiotherapy. Participants were recruited consecutively during the study period, and all men who met the inclusion criteria were invited to participate. The population was divided into two groups according to serum total testosterone levels, using a cutoff value of 2.64 ng/mL, in accordance with the recently published recommendations of the Endocrine Society [13]. Group 0 included men with levels greater than or equal to 2.64 ng/mL, and group 1 included levels less than 2.64 ng/mL. This study was approved by the Ethics Committee of the Ibn Rochd University Hospital Center in Casablanca.

### 2.2. Semen Analysis

Semen samples were collected from participants by masturbation after a period of abstinence of 2–5 days and then liquefied at 37 °C for 30 min in an incubator. Semen analysis was carried out according to the WHO 2021 guidelines [14]. The evaluation included semen volume, sperm concentration, motility, vitality, and morphology.

### 2.3. DNA Fragmentation Index

DFI was determined using the Terminal deoxynucleotidyl transferase-mediated deoxyuridine triphosphate-Nick End Labeling (TUNEL) assay. The principle of this technique was the incorporation of labeled nucleotides into the free ends of 3′DNA fragments in the presence of the terminal deoxynucleotidyl transferase [15]. Semen samples were diluted in phosphate-buffered saline (PBS) and then centrifuged for 10 min. The supernatants were eliminated, and the pellets were smeared onto slides, air-dried, and fixed by immersion in 37 °C formaldehyde prepared in PBS for 30 min. The samples were then rinsed and added to a few drops of permeabilization solution containing Triton X-100, citrate, and distilled water. Then, the samples were incubated at an ambient temperature for 1 min and left to dry. In the dark, the TUNEL reaction mixtures containing fluorescein for in situ cell death detection (Roche Diagnostics GmbH, Mannheim, Germany) were applied to the smears. The slides were then covered with a coverslip and then incubated at 37 °C for 45 min. After incubation, the slides were rinsed, and finally, a few drops of glycerol were added. The evaluation was performed under a fluorescence microscope using ×100 objectives under immersion. The DFI was obtained in percentage from the calculated ratio of fluorescent spermatozoa (considered DNA fragmented) to a total of 100 spermatozoa observed per slide. DFI greater than 15% was considered pathological.

### 2.4. Sperm Decondensation Index

The Sperm Decondensation Index (SDI) was measured using aniline blue staining. The aniline blue binds to lysine residues, which are abundant in histones, thereby staining them [11].

The previously slides sperm smears were stained in aniline blue for 15 min at ambient temperature and then left to dry. The evaluation was performed under a light microscope using ×100 objectives under immersion. The SDI index was obtained in percentage from the calculation of spermatozoa stained dark blue (considered as spermatozoa with immature chromatin) to a total of 100 spermatozoa observed. An SDI index greater than 30% was considered pathological.

### 2.5. Determinations of Serum Sex Hormones

Testosterone, follicle-stimulating hormone (FSH), and LH were analyzed from blood samples provided in the early morning, and then centrifuged to obtain serum. The assay was performed by electrochemiluminescence immunoassay (ECLIA) on a Roche Cobas analyzer. The testosterone was measured using the Elecsys Testosterone II kit (Roche Diagnostics GmbH, Sandhofer Strasse 116, D-68305 Mannheim, Germany) according to the manufacturer’s recommendations. The reference values used for FSH and LH in men were, respectively, from 1.5 to 12.4 UI/L and from 1.7 to 8.6 UI/L.

### 2.6. Statistical Analysis

Statistical analysis of the data was performed using R 4.5.2 software. The normality was evaluated with the Shapiro–Wilk test. Continuous variables were analyzed using Student’s *t*-test or the Mann–Whitney U test, depending on their distribution. The correlation between variables was assessed using the Pearson or Spearman test, depending on their distribution.

## 3. Results

### 3.1. Baseline Characteristic of the Study Population

The study included 214 men, among whom 135 had normal total testosterone levels (≥2.64 ng/mL), and 79 had low levels (<2.64 ng/mL). Table 1 presents the clinical and hormonal characteristics of the participants, including age, FSH and LH concentrations, and total testosterone according to the groups. The mean age of men in the normal testosterone group (group 0) was 39 ± 4.7 years, whereas the group with low testosterone (group 1) was 41 ± 4. This difference was statistically significant with *p* = 0.0015. The levels of FSH and LH were also significantly higher in the low testosterone group (9.38 ± 7.3 UI/L and 7.18 ± 4.18 UI/L, respectively), than in those with normal testosterone (5.75 ± 4.5 UI/L and 4.34 ± 3.69 UI/L; *p* < 0.001 for both). Total testosterone was significantly lower in group 1 than in group 0 (2 ± 0.5 ng/mL vs. 4.66 ± 1.8 ng/mL; *p* < 0.001).

### 3.2. Semen Parameters According to Testosterone Levels

**Figure 1:**
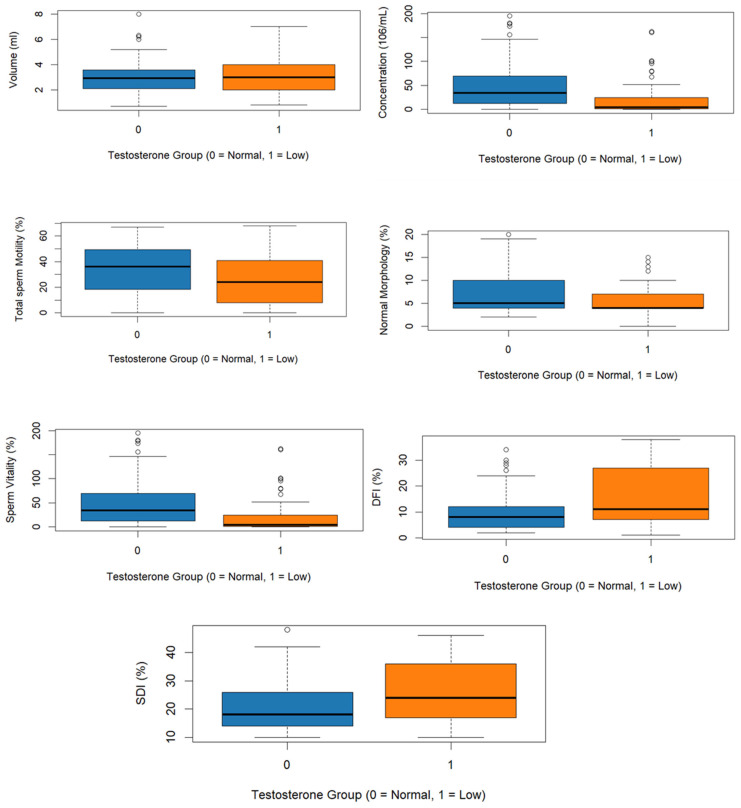
Boxplots of semen parameter, DFI, and SDI according to testosterone levels.

Semen parameters of the two groups are presented in Table 2. The mean semen volume showed no significative difference between the two groups (3.03 ± 1.22 mL vs. 3.10 ± 1.22 mL; *p* = 0.515); however, sperm concentration was significantly lower in the group of low total testosterone compared to the normal total testosterone group (19.6 ± 33.3 × 10^6^/mL vs. 45.8 ± 43.2 × 10^6^/mL; *p* < 0.001). Total motility was also reduced in the low testosterone group (26.2 ± 19.9% vs. 33.7 ± 19.3%; *p* = 0.011). Likewise, normal morphology of spermatozoa (5.78 ± 3.60% vs. 7.69 ± 4.63%; *p* = 0.003) and vitality (60.2 ± 21.2% vs. 66.4 ± 17.8%; *p* = 0.024) were significantly lower in men with low total testosterone (Figure 1).

### 3.3. DFI and SDI According to Testosterone Levels

The DFI and SDI indices of the two groups are presented in Table 3. DFI and SDI were significantly higher in the low testosterone group (15.93 ± 10.82% and 25.8 ± 10.62%, respectively), than in those with normal testosterone (9.99 ± 7.75% and 20.6 ± 8.52%; *p* < 0.001, *p* = 0.001) (Figure 1).

### 3.4. Correlation Between Testosterone and Semen Parameters

**Figure 2:**
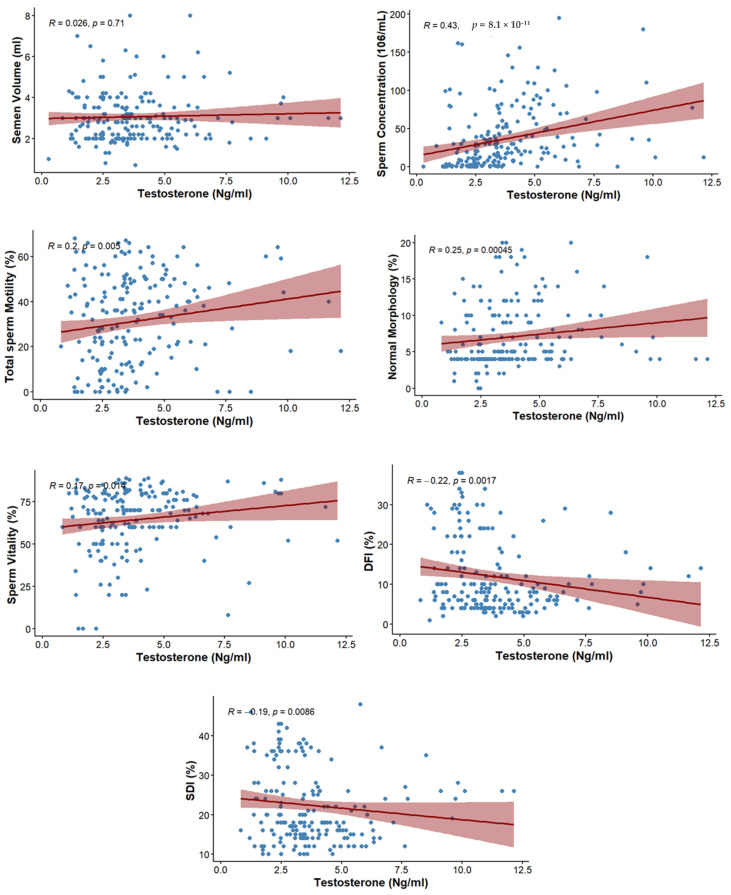
Scatterplots depicting the correlations between testosterone levels and sperm parameters, including DFI, SDI, and conventional semen parameters.

The correlation between testosterone level and semen parameters is presented in Table 4. No significant association was observed with ejaculate volume (rs = 0.026; *p* = 0.710). However, sperm concentration showed a moderate positive correlation with testosterone (rs = 0.43; *p* ≤ 0.001). Total motility (rs = 0.2; *p* = 0.005), normal morphology (rs = 0.25; *p* ≤ 0.001), and vitality of spermatozoa (rs = 0.173; *p* = 0.014) also showed positive correlations with testosterone (Figure 2).

### 3.5. Correlation Between Testosterone and DFI, SDI

The correlation between testosterone level and markers of sperm integrity is presented in Table 5. The level of testosterone was negatively correlated with DFI (rs = −0.221; *p* = 0.0017) as well as SDI (rs = −0.19; *p* = 0.0086) (Figure 2).

## 4. Discussion

The aim of this study was to examine the association between testosterone and sperm DNA fragmentation, chromatin condensation, and conventional semen parameters. A total of 214 men undergoing infertility evaluation were included. The results of the present study indicate that low testosterone levels are associated with increased DNA damage. Furthermore, reduced testosterone levels are linked to global alteration of sperm quality.

Different international guidelines propose variable thresholds for low total testosterone, and no universal cutoff exists due to variations in clinical objectives, populations, and biological factors. The Endocrine Society recommends a cutoff of 2.64 ng/mL (264 ng/dL), harmonized according to CDC standards for young healthy men, which is particularly relevant for evaluating fertility and semen parameters [13]. The European Association of Urology (EAU) suggests a threshold of 12 nmol/L (~3.5 ng/mL) based on meta-analyses showing that testosterone replacement therapy is generally ineffective above this level, with positive effects more pronounced below 12 nmol/L, especially in men with severe hypogonadism (<8 nmol/L) [16]. The American Urological Association (AUA) recommends 3.0 ng/mL (~10.4 nmol/L), based on clinical symptoms and biochemical evaluation, primarily in older or symptomatic men [17]. We chose the Endocrine Society threshold for this study because it is the most appropriate for assessing the impact of testosterone on semen quality in a prospective study of men evaluated for infertility, and our laboratory assays are certified for this cutoff.

This study confirms the role of testosterone in spermatogenesis through a positive correlation between testosterone and sperm concentration, motility, morphology, and vitality. However, no significant association was found between testosterone and semen volume. Consistent with our findings, Keskin et al. reported no association between testosterone and sperm volume, a significant positive association with motility and progressive motility, and a weakly significant association with sperm morphology [18]. Similarly, Trussell et al. demonstrated that low testosterone levels were associated with abnormal sperm morphology [19]. Rehman et al. also reported that low levels of testosterone were observed in participants with impaired semen parameters compared to normospermic men [20]. In contrast, Guardo et al. reported that low total testosterone was not associated with subnormal sperm parameters in infertile couples [7]. However, the retrospective design of their study, the inclusion of only participants with total sperm counts greater than 5 million, and the use of the 2010 guidelines for semen analysis may partly explain the differences compared to our prospective study. Likewise, Zhao et al. observed an inverse association between total testosterone and sperm motility, and no significant association with sperm concentration and morphology [21].

To our knowledge, few studies have specifically investigated the association between serum testosterone levels and the SDI assessed using aniline blue staining. In this context, the present study found a significant negative correlation between testosterone levels and SDI. These findings are supported by experimental studies, which have shown that supplementation with low doses of testosterone in the sperm culture medium has positive effects on chromatin quality [22]. In addition, we observed a significant negative correlation between testosterone and DFI. These associations suggest that low testosterone levels could be related to an increase in sperm damage. These findings are consistent with a recent study reporting an inverse association between serum testosterone and DFI following FSH administration in men with idiopathic infertility [23]. Our results also partially agree with a study of fertile men, which demonstrated a negative association between free testosterone, estradiol, and DFI [24]. In contrast, Appasamy et al. reported that testosterone was not associated with sperm DNA damage [25].

The heterogeneity observed among some previous studies may be explained by several factors, including differences in study design, population characteristics, and other confounding factors. The prospective design of our study represents a key strength, as semen analyses were performed in the same laboratory in accordance with 2021 WHO guidelines, and the natural diurnal variation in serum testosterone was minimized by collecting blood samples early in the morning. Combined with the evaluation of both conventional semen parameters and advanced markers of sperm integrity, as well as sperm vitality, which has been rarely studied, these methodological approaches may explain the more consistent association observed between testosterone levels and sperm quality in our population. Our results, therefore, add to the evidence and help further clarify this relationship in a North African population that is still poorly documented.

Testosterone acts on Sertoli cells, promoting their proliferation and maturation, which are essential for germ cell development. This process enables the production of mature, intact spermatozoa capable of fertilization. Studies have shown that the absence of androgen signaling results in the arrest of Sertoli cell maturation [26], highlighting the central role of testosterone in maintaining functional spermatogenesis and overall sperm quality. The effect of testosterone is not limited to regulating the intrinsic genomic activity of Sertoli cells but also influences germ cells through paracrine signaling. Testosterone participates in the self-renewal and differentiation of germ cells. Classical and non-classical testosterone signaling, mediated by androgen receptors (AR) in Sertoli cells, is necessary for normal meiosis [26], which allows the production of functional spermatozoa with normal morphology and motility. Moreover, in SCARKO mice, in which the classical signaling pathway is blocked, meiosis arrest occurs at the prophase I [26].

In our study, we observed a negative correlation between serum testosterone levels and sperm DNA fragmentation as well as chromatin decondensation. These findings can be explained by known biological mechanisms. In the absence of AR signaling, germ cells initiate prophase I of meiosis normally and undergo double-strand break formation. However, anomalies arise during the repair of these double-strand breaks and during chromosome synapsis [27,28], leading to chromatin instability. Furthermore, androgen deprivation has been associated with decreased expression of genes encoding protective proteins against oxidative stress, such as Aldh2, Prdx6, and Gstm5, which increases oxidative stress in germ cells [29]. This oxidative stress, in turn, induces DNA fragmentation, compromising the integrity of genetic information and potentially impairing male fertility [30].

A decrease in androgen levels has been associated with increased expression of ubiquitin carboxyl-terminal hydrolase in spermatocytes, which promotes the déubiquitination of the pro-apoptotic p53. Activation of p53 subsequently triggers apoptosis of germ cells, leading to a reduction in sperm production [31]. In addition, testosterone strengthens the adhesion of spermatids to Sertoli cells. When this adhesion is disrupted, spermatids detach prematurely, fail to complete their maturation, and are subsequently phagocytosed by Sertoli cells [26,32,33]. These actions of testosterone indicate the positive association observed in our study between testosterone levels and sperm concentration. Moreover, testosterone indirectly stimulates the production of plasma membrane Ca^2+^ ATPase 4 in spermatids. This protein helps regulate intracellular calcium, which is essential for sperm motility at the time of their release [34].

This study has some limitations. The absence of some key parameters, such as SHBG and free testosterone, would have allowed for a more comprehensive endocrine interpretation. Furthermore, Semen parameters vary within the same individual over time, and a single semen sample may not reliably reflect a man’s long-term values.

## 5. Conclusions

In conclusion, our study reports a significant positive correlation between serum total testosterone levels and conventional semen parameters, including sperm concentration, motility, morphology, and vitality, as well as a significant negative correlation with DNA fragmentation and chromatin decondensation. These results indicate that lower testosterone levels were associated with alterations in semen quality. These findings support the essential role of testosterone in sustaining spermatogenesis, semen quality, and sperm DNA integrity and highlight the crucial importance of testosterone assessment in the diagnosis and pathophysiological understanding of male infertility. Further investigations could evaluate whether correcting testosterone levels in men below the identified threshold could have an impact on sperm DNA integrity, semen parameters, and reproductive outcomes.

## Figures and Tables

**Table 1:** Comparison of baseline characteristics of the study population between the low total testosterone group and the normal total testosterone group.

	Group 0 (TT ≥ 2.64 ng/mL)	Group 1 (TT < 2.64 ng/mL)	*p*-Value
Age (years)			0.0015 ^y^
Mean (SD)	39 ± 4.7	41 ± 4	
Median (IQR)	39 (36–43)	41 (38–45)	
FSH (UI/L)			<0.001 ^x^
Mean (SD)	5.75 ± 4.5	9.38 ± 7.3	
Median (IQR)	4.40 (3.14–6.63)	7.80 (4.05–13.05)	
LH (UI/L)			<0.001 ^x^
Mean (SD)	4.34 ± 3.69	7.18 ± 4.18	
Median (IQR)	3 (2–5.71)	7.12 (4.10–10)	
TT (ng/mL)			<0.001 ^x^
Mean (SD)	4.66 ± 1.8	2 ± 0.5	
Median (IQR)	4.02 (3.42–5.29)	2.12 (1.67–2.48)	

Values are expressed as mean ± standard deviation (SD) and median with interquartile range (IQR) (25–75%). ^x^: Mann–Whitney test. ^y^: Student’s *t*-test. FSH: follicle-stimulating hormone (UI/L). LH: luteinizing hormone (UI/L). TT: total testosterone (ng/mL).

**Table 2:** Comparison of semen parameters between the low total testosterone group and the normal total testosterone group.

	Group 0 (TT ≥ 2.64 ng/mL)	Group 1 (TT < 2.64 ng/mL)	*p*-Value
Semen Volume (mL)			0.515
Mean (SD)	3.03 ± 1.22	3.10 ± 1.22	
Median (IQR)	2.90 (2.10–3.60)	3 (2–3.4)	
Sperm Concentration (10^6^/mL)			<0.001
Mean (SD)	45.8 ± 43.2	19.6 ± 33.3	
Median (IQR)	34 (12–69.5)	4 (0.8–25)	
Total sperm Motility (%)			0.011
Mean (SD)	33.7 ± 19.3	26.2 ± 19.9	
Median (IQR)	36 (18.50–49.5)	24 (8–41)	
Normal Morphology (%)			0.003
Mean (SD)	7.69 ± 4.63	5.78 ± 3.60	
Median (IQR)	5 (4–10)	4 (4–7)	
Sperm Vitality (%)			0.024
Mean (SD)	66.4 ± 17.8	60.2 ± 21.2	
Median (IQR)	70 (60–80)	64 (50–77)	

Values are expressed as mean ± standard deviation (SD) and median with interquartile range (IQR) (25–75%).

**Table 3:** Comparison of DFI and SDI between the low total testosterone group and the normal total testosterone group.

	Group 0 (TT ≥ 2.64 ng/mL)	Group 1 (TT < 2.64 ng/mL)	*p*-Value
DFI (%)			<0.001
Mean (SD)	9.99 ± 7.75	15.93 ± 10.82	
Median (IQR)	8 (4–12)	11 (7–26.5)	
SDI (%)			0.001
Mean (SD)	20.6 ± 8.52	25.8 ± 10.62	
Median (IQR)	18 (14–26)	24 (17.5–36)	

Values are expressed as mean ± standard deviation (SD) and median with interquartile range (IQR) (25–75%).

**Table 4:** Spearman’s rank correlation coefficient (rs) between testosterone and semen parameters.

	Spearman’s Rank with Testosterone	*p*-Value
Semen Volume (mL)	0.026	0.710
Sperm Concentration (10^6^/mL)	0.43	<0.001
Total sperm Motility (%)	0.2	0.005
Normal Morphology (%)	0.25	<0.001
Sperm Vitality (%)	0.173	0.014

**Table 5:** Spearman’s range correlation coefficient between testosterone, DFI, and SDI.

	Spearman’s Rank with Testosterone	*p*-Value
DFI (%)	−0.221	0.0017
SDI (%)	−0.19	0.0086

## Data Availability

The datasets supporting the conclusions of this article are included within the article.

## Abbreviations

The following abbreviations are used in this manuscript:

WHO	World Health Organization
LH	Luteinizing Hormone
GnRH	Gonadotropin-Releasing Hormone
SDF	Sperm DNA Fragmentation
DFI	DNA Fragmentation Index
SCC	Sperm Chromatin Condensation
TUNEL	Terminal deoxynucleotidyl transferase-mediated deoxyuridine triphosphate-Nick End Labeling.
PBS	Phosphate-Buffered Saline
SDI	Sperm Decondensation Index
FSH	Follicle-stimulating hormone
ECLIA	Electrochemiluminescence immunoassay
TT	Total Testosterone
SD	Standard deviation
IQR	Median with interquartile range
rs	Spearman’s rank correlation coefficient
AR	Androgen receptors
EAU	European Association of Urology
AUA	American Urological Association
